# Comparison of the clinical outcomes after esophagectomy between intrathoracic anastomosis and cervical anastomosis: a systematic review and meta-analysis

**DOI:** 10.1186/s12893-022-01875-7

**Published:** 2022-12-08

**Authors:** Qi-Yue Ge, Yu-Heng Wu, Zhuang-Zhuang Cong, Yong Qiang, Yan-Qing Wang, Chao Zheng, Yi Shen

**Affiliations:** 1 Department of Cardiothoracic Surgery, Jinling Hospital, School of Medicine, Southeast University, Nanjing, China; 2 Department of Thoracic Surgery, National Cancer Center/National Clinical Research Center for Cancer/Cancer Hospital, Chinese Academy of Medical Sciences and Peking Union Medical College, Beijing, 100021 China; 3 Department of Cardiothoracic Surgery, Jinling Hospital, Medical School of Nanjing University, Nanjing, China; 4 Department of Cardiology, Jinling Hospital, School of Medicine, Medical School of Nanjing University, Nanjing, China

**Keywords:** Esophagectomy, Intrathoracic anastomosis, Cervical anastomosis, Clinical outcomes, Meta-analysis

## Abstract

**Objectives:**

Esophageal cancer is a high-mortality disease. Esophagectomy is the most effective method to treat esophageal cancer, accompanied with a high incidence of post-operation complications. The anastomosis has a close connection to many severe post-operation complications. However, it remains controversial about the choice of intrathoracic anastomosis (IA) or cervical anastomosis (CA). The study was conducted to compare the clinical outcomes between the two approaches.

**Methods:**

We searched databases for both randomized controlled trials (RCTs) and cohort studies comparing post-operation outcomes between IA and CA. Primary outcomes were the incidences of anastomotic leakage and mortality. Secondary outcomes were the incidences of anastomotic stenosis, pneumonia and re-operation.

**Results:**

Twenty studies with a total of 7,479 patients (CA group: n = 3,183; IA group: n = 4296) were included. The results indicated that CA group had a higher incidence of anastomotic leakage than IA group (odds ratio [OR] = 2.05, 95% confidence intervals [CI] = 1.61–2.60, I^2^ = 53.31%, P < 0.01). Subgroup analyses showed that CA group had higher incidences of type I (OR = 2.19, 95%CI = 1.05–4.57, I^2^ = 0.00%, P = 0.04) and type II (OR = 2.75, 95%CI = 1.95–3.88, I^2^ = 1.80%, P < 0.01) anastomotic leakage than IA group. No difference was found in type III anastomotic leakage (OR = 1.23, 95%CI = 0.82–1.86, I^2^ = 20.92%, P = 0.31). The 90-day mortality (OR = 1.66, 95%CI = 1.11–2.47, I^2^ = 0.0%, P = 0.01) in IA group were lower than that in CA group. No difference was found in in-hospital mortality (OR = 1.31, 95%CI = 0.91–1.88, I^2^ = 0.00%, P = 0.15) and 30-day mortality (OR = 1.08, 95%CI = 0.69–1.70, I^2^ = 0.00%, P = 0.74).

**Conclusions:**

IA might be a better anastomotic approach than CA, with a lower incidence of anastomosis leakage and no increase in short-term mortality. Significant heterogeneity and publication bias might limit the reliability of the results. More high-quality studies are needed to verify and update our findings.

## Introduction

Esophageal cancer has been reported to be the sixth high-mortality and the seventh high-incidence cancer in 2020 [1]. For early-staged esophageal cancer, esophagectomy could be a preferred treatment strategy. However, the complexity of the operation also has a high risk to trigger complications [2].

Since esophagectomy was firstly reported in 1913 [3], the esophagectomy operation has experienced several evolutions. During the surgery, the stomach needs to be made into a conduit, and then to be anastomosed with the rest of esophagus [2]. The anastomosis can be made either in the chest or in the neck, concerning to the location of tumor and the preference of surgeon. A heated debate of the location of anastomosis has lasted for several years. Surgeons prefer intrathoracic anastomosis to cervical anastomosis due to its lower leakage rate. Others believe the cervical anastomosis is a better choice owing to its lower leak-related mortality. Previous study compared Ivor-Lewis approach with McKeown approach [4], which demonstrated that Ivor-Lewis approach was a better option. As Ivor-Lewis is one of the esophagectomy approaches with IA and McKeown is one of the esophagectomy approaches with CA, IA might be better than CA. However, no more detailed analysis on anastomotic leakage was done since anastomotic leakage is one of the most important post-operation complications. Thus, we launch this study to compare cervical anastomosis and intrathoracic anastomosis, in terms of the severe complications, especially anastomotic leakage, and mortality.

## Methods

### Registration

This research satisfied the preferred reporting items for systematic reviews and meta-analysis (PRISMA) [5]. The protocol of the systematic review and meta-analysis was registered in PROSPERO (CRD42022300258).

### Eligibility criteria

The study incorporated into the systematic review must satisfy PICOS criteria as follow:

P(Patients): Male or Female patients underwent esophagectomy.

I(Intervention): Any kinds of esophagectomy with cervical anastomosis.

C(Control): Any kinds of esophagectomy with intrathoracic anastomosis.

O(Outcome): Anastomosis Leak or Mortality must be included.

S(Study): RCTs and cohort studies.

### Exclusion criteria

The exclusion criteria were listed as following: (1) duplicate studies; (2) studies without comparison between IA and CA; (3) non-English literature;

### Search

PubMed, Web of Science and ClinicalTrials.gov were searched via the following strategy: (esophagectomy [MeSH] OR esophagus [MeSH] OR oesophagus [Title/Abstract] OR esophagus [Title/Abstract] OR oesophageal [Title/Abstract] OR esophageal [Title/Abstract] OR oesophagectomy [Title/Abstract] OR esophage* [Title/Abstract]) AND (anastomosis, surgical [MeSH] OR anastomo*) AND (intrathoracic OR intra-thoracic OR thoracic OR Ivor Lewis OR Ivor-Lewis OR transthoracic OR trans-thoracic) AND (cervical OR McKeown OR transhiatal OR trans-hiatal) AND ("2001/01/01"[Date—Publication]: "2022/04/25"[Date—Publication]).

### Study selection

The screening of the title and abstract was performed independently by two reviewers (Qi-Yue Ge and Yu-Heng Wu) using PICOS criteria. In the first stage, two reviewers selected the studies from 1^st^ January 2001 to 25^th^ April 2022 by the title and abstract independently. Then, the full texts of the studies selected in the first stage were estimated by the two reviewers to determine whether the studies meet the inclusion criteria. Any disagreement of the inclusion was recorded and discussed in the review team. The decision was ultimately made by a third member of the review team (Chao Zheng).

### Data collection

The data were collected by two reviewers (Qi-Yue Ge, Yu-Heng Wu) independently using predefined sheet. If any difference exists, the controversial data will be confirmed by a third reviewer (Chao Zheng).

### Outcome indicators

Main outcomes (anastomotic leak and mortality) and secondary outcomes (reoperation, other complications: anastomotic stenosis and pneumonia).

### Data included

General information (published year, author and published journal), participant characteristics of the study (age, gender, and neoadjuvant treatment) and in-operation information (operation time and blood loss in operation).

### Risk of Bias

The risk of bias of each study was independently evaluated by two reviewers (Qi-Yue Ge and Yu-Heng Wu). The RCTs were evaluated by Jaded scale [6] and the cohort study were evaluated by NOS [7].

### Statistical analysis

According to the PRISMA guidelines, data analyses were done by STATA 16.0 software (Stata Corp, College Station, TX, USA). The difference of clinical outcomes between CA and IA was described by forest plots using fixed-effected inverse-variance model and the random effect model will be employed according to the heterogeneity (if I^2^ ≥ 50%). The result would be considered as statistically significance if the P value was less than 0.05. The comparison was done by pooled ORs with 95% CIs. The heterogeneity was assessed by Chi-squared using Q statistics and I^2^ test. Sensitivity analyses were applied to find the source of heterogeneity if necessary (I^2^ ≥ 50% or P < 0.05). The publication bias was assessed by funnel plots and L’Abbe plots and if necessary, Egger’s test would be done.

## Results

### Study characteristics

**Fig. 1:**
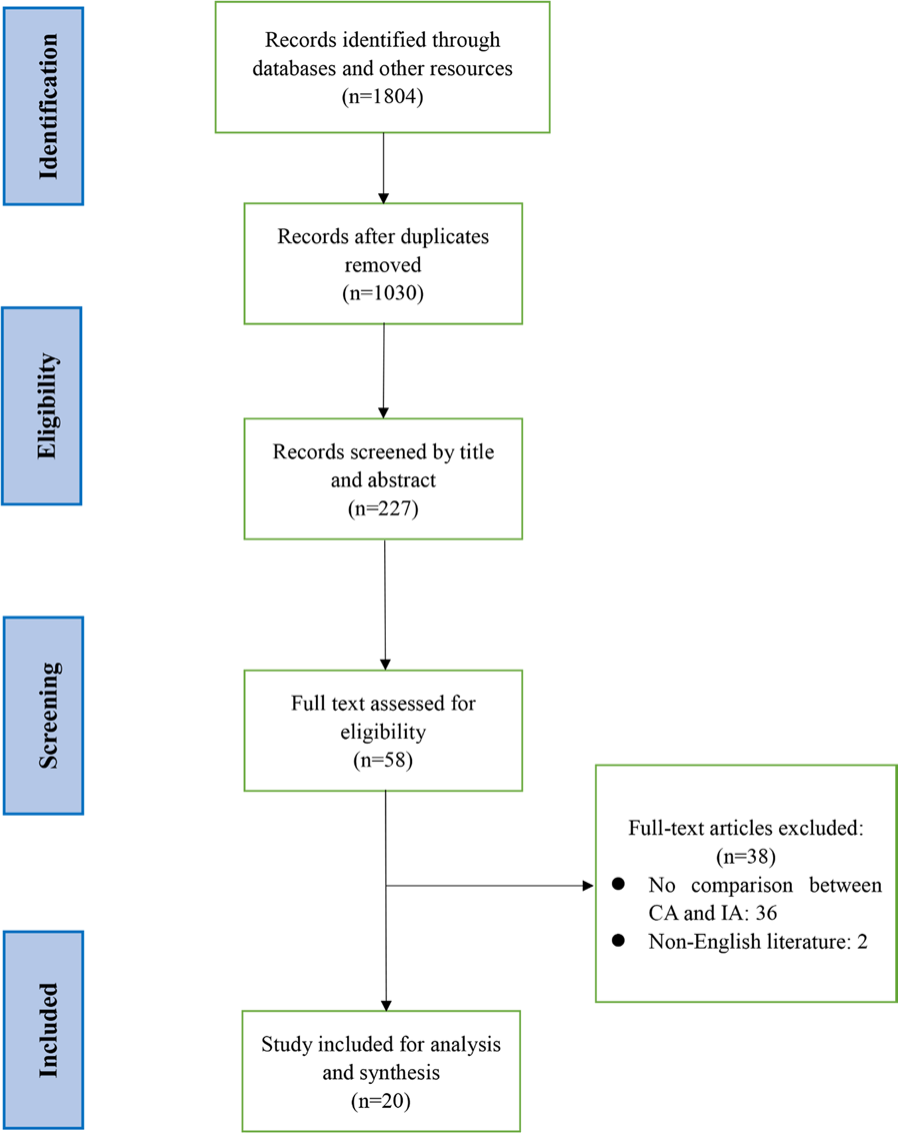
Flow chart of selection for included studies

Literature search and study selection were shown in Fig. 1. A total of 1,208 potential studies were systematically searched from PubMed, Web of Science and ClinicalTrials.gov. Twenty of them were finally included after screening and exclusions (Fig. 1). The basic characteristics of the included studies were shown in Table 1 and 7479 patients (CA group: n = 3183; IA group: n = 4296) were included. Three RCTs assessed by Jaded scale were presented in Table 2. Seventeen cohort studies evaluated by NOS were shown in Table 3.

**Table 1:** Characteristics of the selected studies

year	Country	author	study design	Group	N	Neoadjuvant	Tumor location (U/M/L/J/O)	Anastomotic technique (HS/S/O)	Tumor pathology (AC/SC/O)	Anastomotic leakage
2001	Switzerland	Schilling et al. [14]	Cohort study	CA	62	NA	NA	0/62/0	12/37/13	5
				IA	33	NA	NA	0/33/0	30/1/2	2
2001	Canada	Blewett et al. [15]	Cohort study	CA	19	NA	NA	NA	11/8/0	1
				IA	55	NA	NA	NA	40/15/0	9
2003	Sweden	Walther et al. [16]	RCT	CA	41	NA	3/19/16/0/1	41/0/0	14/25/2	1
				IA	42	NA	1/10/24/0/0	0/42/0	18/17/7	0
2006	Yemen	Homesh et al. [17]	Cohort study	CA	43	NA	6/2/34/1/0	NA	24/19/0	9
				IA	41	NA	3/16/18/4/0	NA	23/18/0	5
2007	Japan	Okuyama et al. [18]	RCT	CA	18	NA	0/13/5/0/0	18/0/0	0/17/1	3
				IA	14	NA	0/10/4/0/0	0/14/0	0/13/1	1
2008	Germany	Egberts et al. [19]	Cohort study	CA	33	15	NA	NA	3/29/1	11
				IA	72	29	NA	NA	41/25/6	13
2011	India	Kawoosa et al. [20]	Cohort study	CA	205	NA	0/91/71/33/10	NA	91/98/16	23
				IA	177	NA	0/81/67/23/6	NA	58/105/14	5
2012	Germany	Klink et al. [21]	Cohort study	CA	36	NA	NA	36/0/0	26/10/0	11
				IA	36	NA	NA	0/36/0	29/7/0	4
2015	China	Zhai et al. [22]	Cohort study	CA	40	NA	0/23/17/0/0	0/40/0	6/32/2	12
				IA	32	NA	0/15/17/0/0	0/32/0	5/26/1	3
2015	China	Huang et al. [23]	Cohort study	CA	114	NA	0/114/0/0/0	0/114/0	0/114/0	10
				IA	91	NA	0/91/0/0/0	0/91/0	0/91/0	2
2016	Netherlands	Workum et al. [24]	Cohort study	CA	146	137	0/0/106/40/0	123/5/18	120/23/3	43
				IA	210	201	0/0/172/38/0	6/204/0	189/19/2	43
2017	China	Liu et al. [25]	Cohort study	CA	126	62	0/0/37/89/0	45/32/49	90/28/2	21
				IA	332	176	0/0/64/268/0	33/201/98	249/77/6	34
2018	Netherlands	Gooszen et al. [26]	Cohort study	CA	654	600	0/47/607/0/0	NA	533/104/17	143
				IA	654	604	0/42/612/0/0	NA	545/92/17	111
2018	China	Shao et al. [27]	Cohort study	CA	282	NA	25/201/56/0/0	NA	0/282/0	42
				IA	282	NA	15/220/47/0/0	NA	0/282/0	12
2019	Germany	Schroder et al. [28]	Cohort study	CA	430	281	NA	175/255/0	289/141/0	74
				IA	536	420	NA	0/536/0	466/70/0	85
2020	America	Chidi et al. [29]	Cohort study	CA	380	380	NA	NA	280/44/56	54
				IA	528	528	NA	NA	369/40/119	65
2020	Netherlands	Workum et al. [30]	Cohort study	CA	210	195	0/0/194/16/0	NA	161/38/11	59
				IA	210	194	0/0/192/18/0	NA	183/24/3	29
2020	Netherlands	Jezerskyte et al. [31]	Cohort study	CA	89	3	0/0/75/14/0	NA	60/24/4	22
				IA	115	22	0/0/98/17/0	NA	103/9/3	10
2021	Netherlands	Workum et al. [8]	RCT	CA	123	120	0/3/106/14/0	108/15/0	114/7/2	42
				IA	122	120	0/6/105/11/0	4/118/0	105/12/5	15
2021	America	Takahashi et al. [32]	Cohort study	CA	132	78	NA	NA	107/19/6	13
				IA	714	459	NA	NA	611/73/30	31

Tumor location (U: Upper/M: Middle/L: Lower/J: Junction/O: other); Anastomotic technique (HS: handsewn/S: Stapler/O: Other); Tumor pathology (AC: Adenocarcinoma/SC: Squamous carcinoma/O: Other)

**Table 2:** The Jadad scale

Study	Random			Blinding			Lost/Exit		Total
	0	1	2	0	1	2	0	1	
Walther et al., 2003		+		+				+	2
Okuyama et al., 2007		+		+			+		1
Workum et al., 2021			+	+				+	3

**Table 3:** The Newcastle–Ottawa scale

Study	Selection				Comparability	Exposure			Quality score
	1	2	3	4	1	1	2	3	
Schilling et al., 2001	★	☆	☆	★	★☆	★	★	★	6
Blewett et al., 2001	★	★	☆	★	★☆	★	★	★	7
Homesh et al., 2006	★	★	☆	★	★☆	★	★	☆	6
Egberts et al., 2008	★	★	☆	★	★☆	★	★	★	7
Kawoosa et al., 2011	★	★	☆	★	★☆	★	★	★	7
Klink et al., 2012	★	★	☆	★	★★	★	★	★	8
Zhai et al., 2015	★	★	☆	★	★☆	★	★	★	7
Huang et al., 2015	★	★	☆	★	★☆	★	★	★	7
Workum et al., 2016	★	★	☆	★	★☆	★	★	★	7
Liu et al., 2017	★	★	☆	★	★☆	★	★	★	7
Gooszen rt al, 2018	★	★	☆	★	★★	★	★	★	8
Shao et al., 2018	★	★	☆	★	★★	★	★	★	8
Schroder et al., 2019	★	★	☆	★	★☆	★	★	★	7
Chidi et al., 2020	★	☆	☆	★	★☆	★	★	★	6
Workun et al., 2020	★	★	☆	★	★★	★	★	★	8
Jezerskyte et al., 2020	★	★	☆	★	★☆	★	★	★	7
Takahashi et al., 2021	★	☆	☆	★	★☆	★	★	★	6

### Primary outcome

#### Anastomotic leakage

**Fig. 2:**
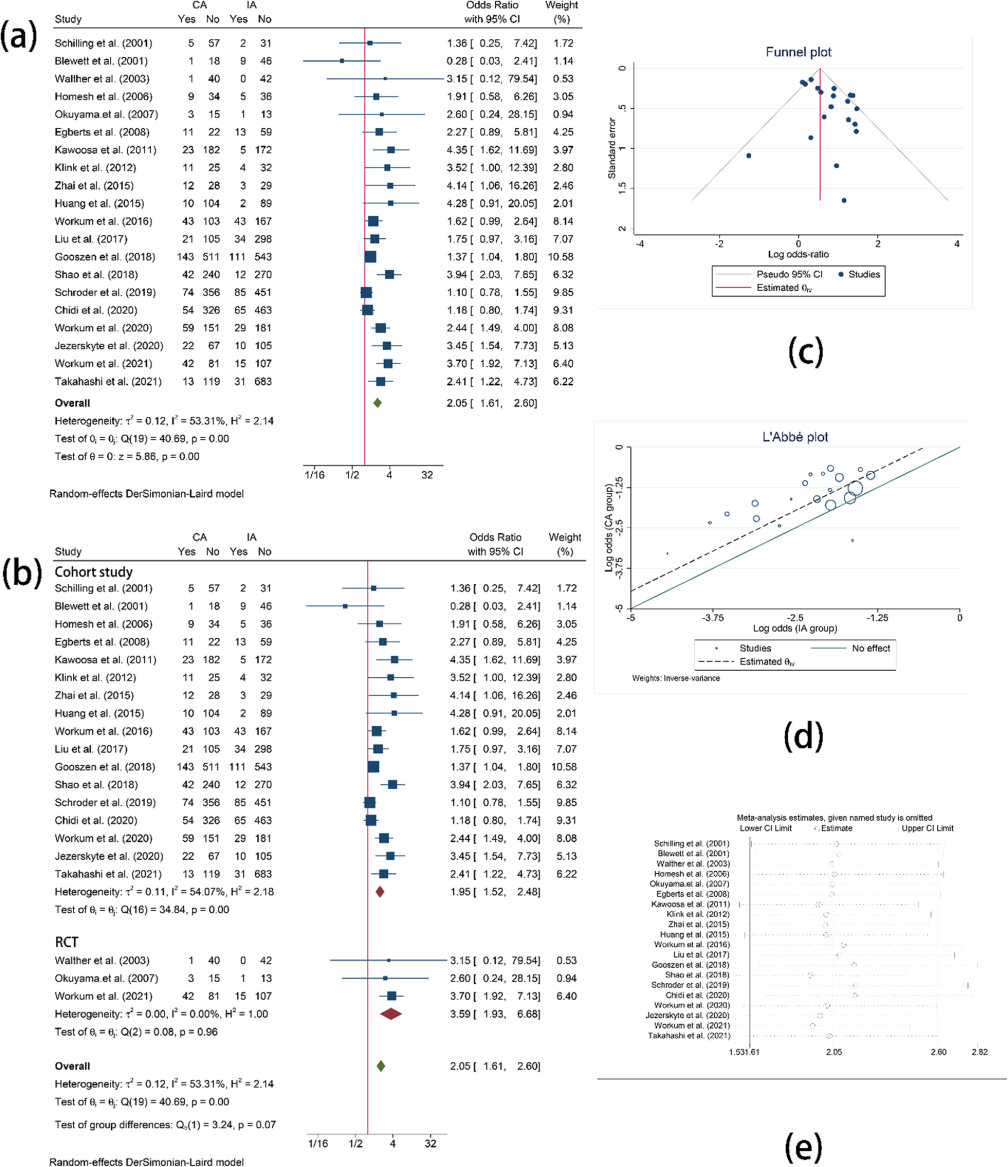
Comparison of the anastomotic leakage. **a**, **b** Comparison of the anastomotic leakage between CA and IA; **c** Funnel plot for anastomotic leakage; **d** L’Abbe plot for anastomotic leakage; **e** Sensitivity analysis for anastomotic leakage

Incidence of anastomotic leakage was reported in all 20 studies. IA was associated with a lower leak rate (OR = 2.05, 95%CI = 1.58–2.65, I^2^ = 68.0%, P < 0.01) (Fig. 2). The result turned to be same when the studies were classified in terms of RCT (OR = 3.59, 95%CI = 1.93–6.68, I^2^ = 0.0%, P < 0.01) or cohort study (OR = 1.97, 95%CI = 1.61–2.60, I^2^ = 53.31%, P < 0.01). Funnel plot, L’Abbe plot and sensitivity analysis were also presented in Fig. 2. As shown in the sensitivity analysis, the result remained significant difference given any one of these studies was omitted.

**Fig. 3:**
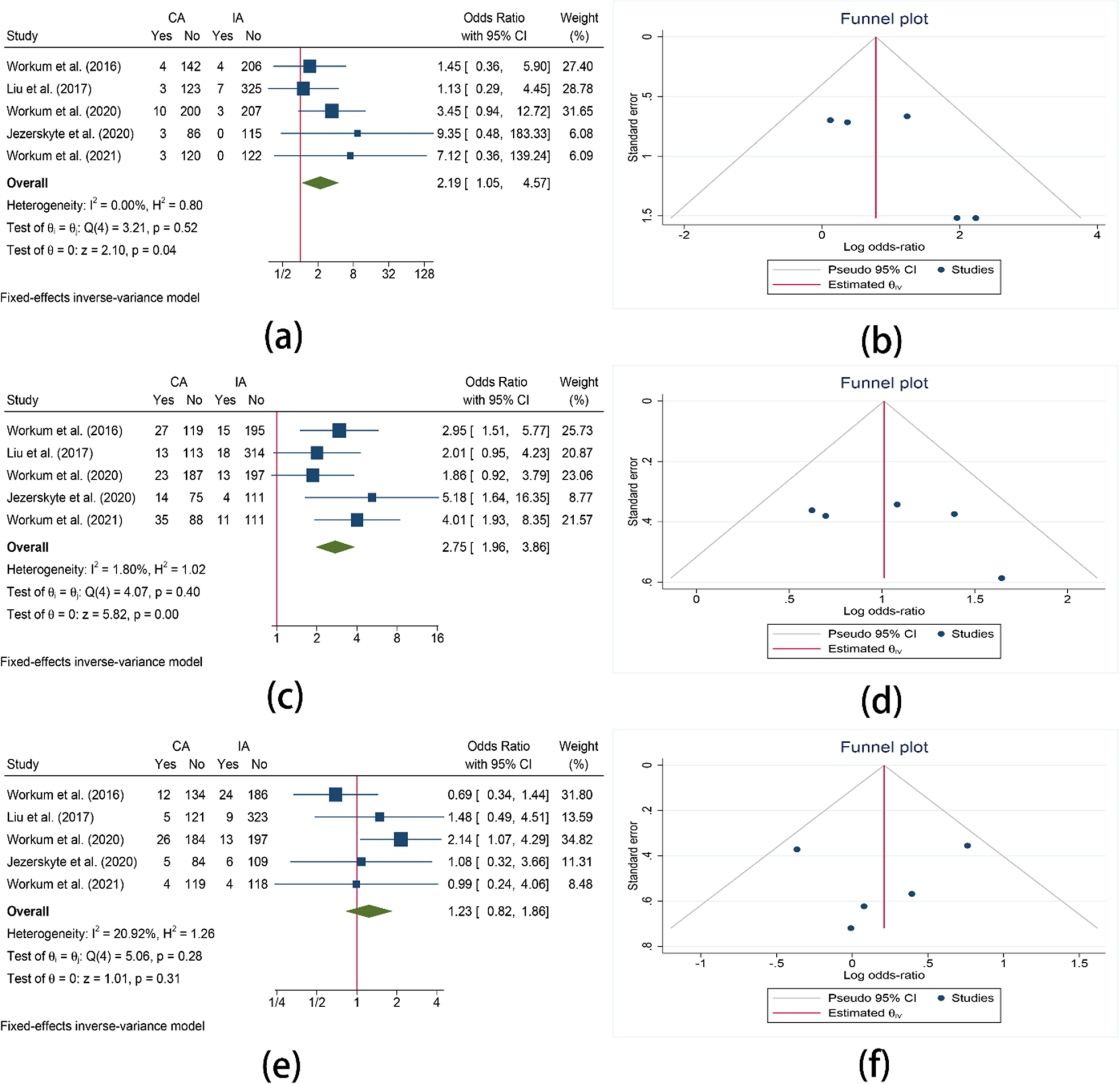
Subgroup analysis of anastomotic leakage. **a** Comparison of the type I anastomotic leakage between CA and IA; **b** Funnel plot for type I anastomotic leakage; **c** Comparison of the type II anastomotic leakage between CA and IA; **d** Funnel plot for type II anastomotic leakage; **e** Comparison of the type III anastomotic leakage between CA and IA; **f** Funnel plot for type III anastomotic leakage

Anastomotic leakage classified by Esophagectomy Complications Consensus Group (ECCG) classification was reported in 5 studies. The forest plots indicated that patients undergoing cervical anastomosis are more likely to suffer from type I (OR = 2.19, 95%CI = 1.05–4.57, I^2^ = 0.00%, P = 0.04) or type II (OR = 2.75, 95%CI = 1.96–3.86, I^2^ = 1.80%, P < 0.01) anastomotic leakage. However, no significant difference was found in type III anastomotic leakage (OR = 1.23, 95%CI = 0.82–1.86, I^2^ = 20.92%, P = 0.31) (Fig. 3).

#### Mortality

**Fig. 4:**
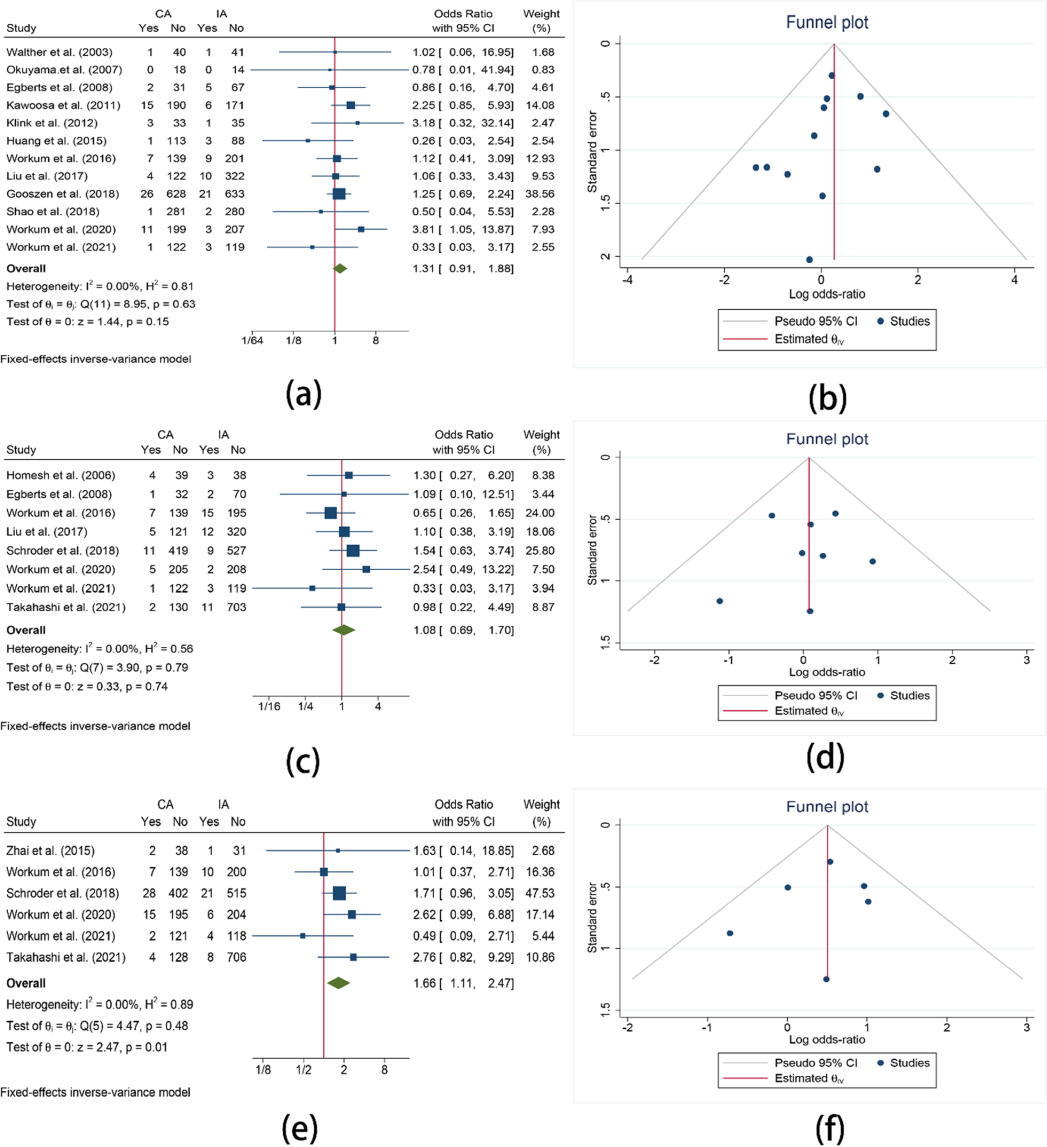
Comparison of the mortality. **a** Comparison of the in-hospital mortality between CA and IA; **b** Funnel plot for in-hospital mortality; **c** Comparison of the 30-day mortality between CA and IA; **d** Funnel plot for 30-day mortality; **e** Comparison of the 90-day mortality between CA and IA; **f** Funnel Plot for 90-day mortality

Mortality was reported in 15 studies. 12 of them reported the in-hospital mortality which demonstrated that no significant difference exists between two approaches (OR = 1.31, 95%CI = 0.91–1.88, I^2^ = 0.00%, P = 0.15). The 30-day mortality reported in 8 studies was also of no significant difference between two approaches (OR = 1.08, 95%CI = 0.69–1.70, I^2^ = 0.00%, P = 0.74). The 90-day mortality rate reported in 6 studies showed that 90-day mortality in IA was significantly lower than that in CA (OR = 1.66, 95%CI = 1.11–2.47, I^2^ = 0.00%, P = 0.01) (Fig. 4).

### Secondary outcome

#### Anastomotic stenosis

**Fig. 5:**
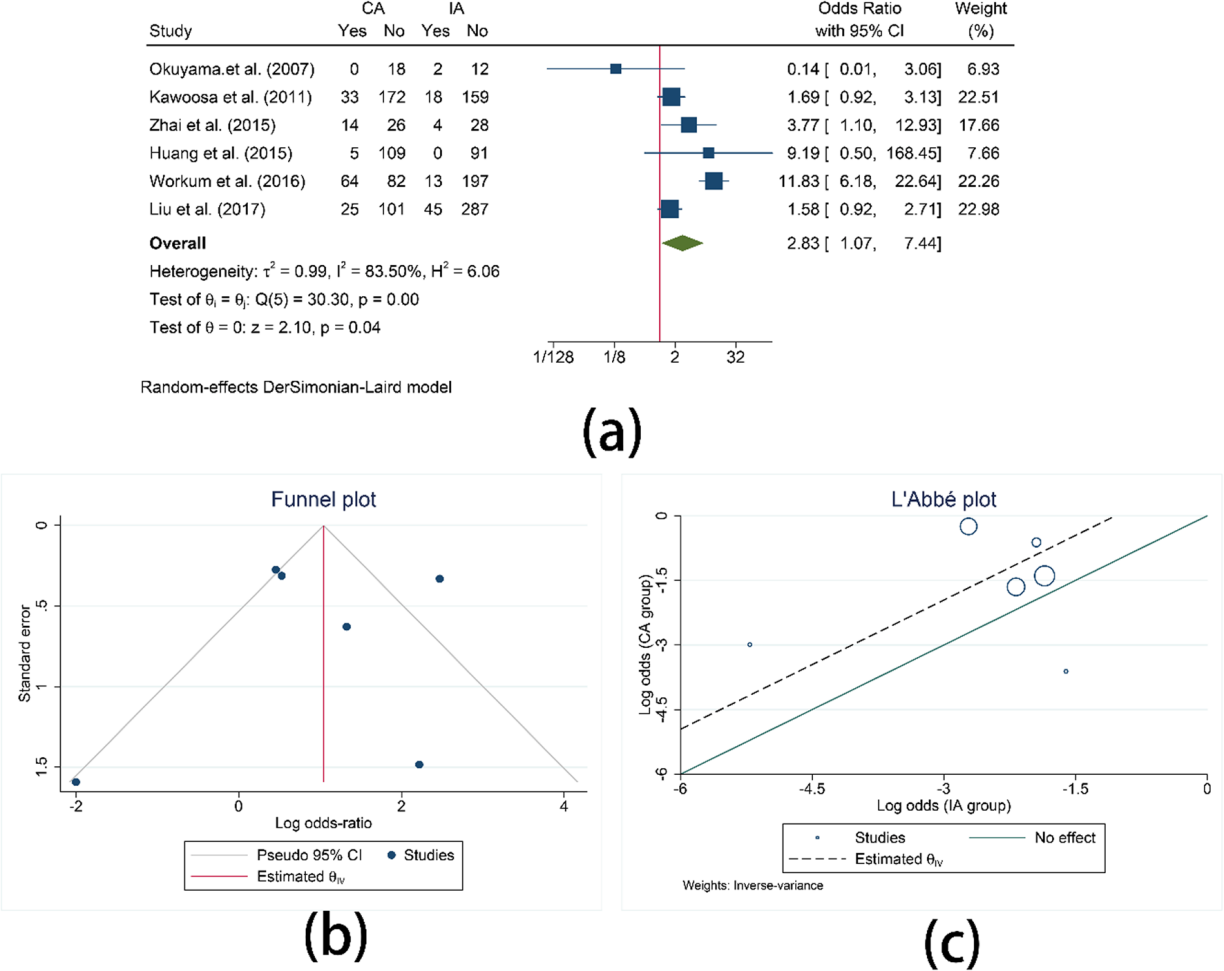
Comparison of the anastomotic stenosis. **a** Comparison of the anastomotic stenosis between CA and IA; **b** Funnel plot for anastomotic stenosis; **c** L’Abbe plot for anastomotic stenosis

Incidence of anastomotic stenosis was reported in 6 studies. The results indicated that patients who underwent intrathoracic anastomosis were less likely to suffer from anastomotic stenosis than that in cervical anastomosis (OR = 2.83, 95%CI = 1.07–7.44, I^2^ = 83.50%, P = 0.04). (Fig. 5). Heterogeneity may exist among studies.

#### Pneumonia

**Fig. 6:**
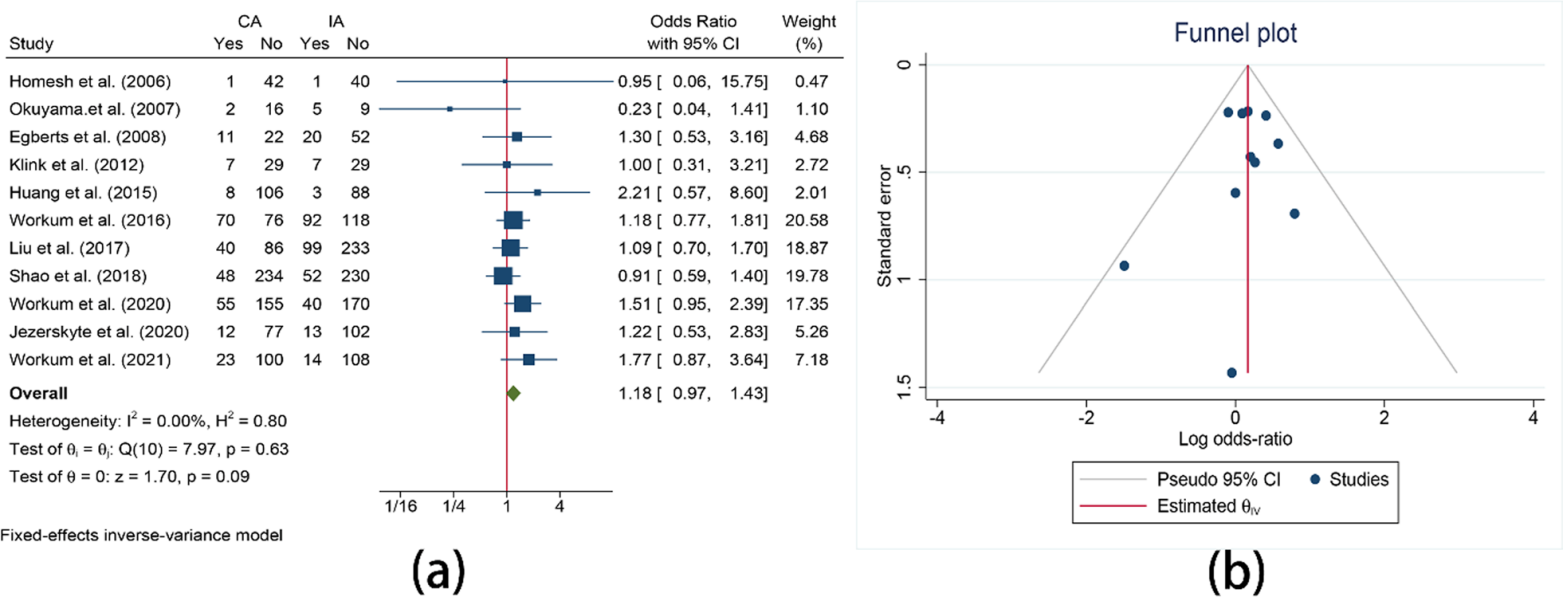
Comparison of the pneumonia. **a** Comparison of the pneumonia between CA and IA; **b** Funnel plot for pneumonia

Incidence of pneumonia was reported in 11 studies. The result showed that no significant difference was found between cervical anastomosis and intrathoracic anastomosis (OR = 1.18, 95%CI = 0.97–1.43, I^2^ = 0.00%, P = 0.09) (Fig. 6).

#### Reoperation

**Fig. 7:**
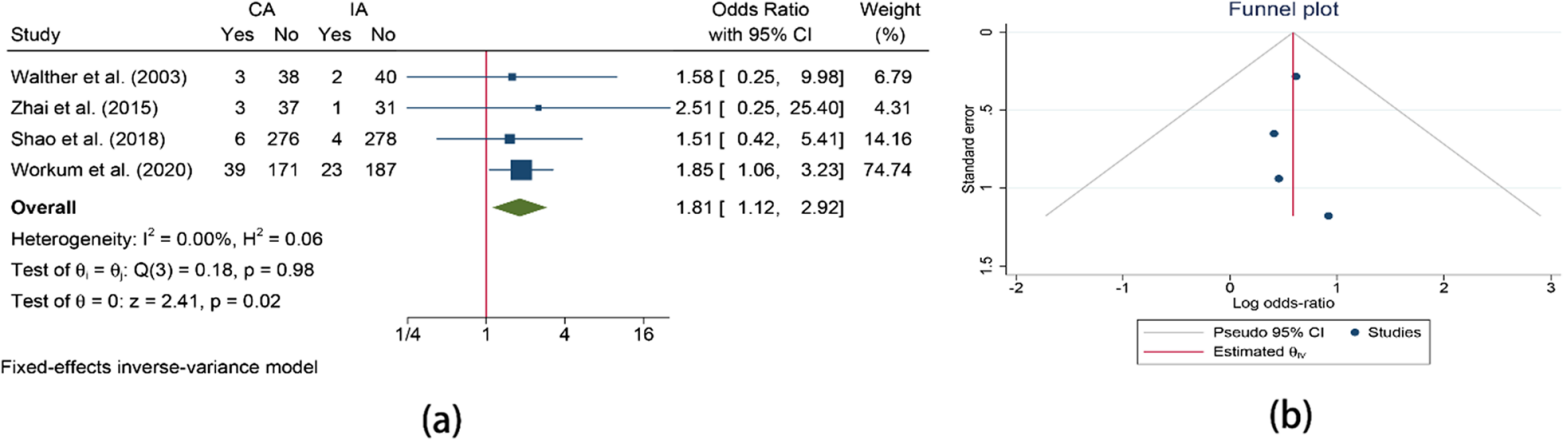
Comparison of the reoperation. **a** Comparison of the reoperation between CA and IA; **b** Funnel plot for reoperation

Reoperation rate was reported in 4 studies. IA had a lower reoperation rate than CA (OR = 1.81, 95%CI = 1.12–2.92, I^2^ = 0.00%, P = 0.02) (Fig. 7).

## Discussion

The systematic review and meta-analysis focused on two anastomotic approaches: IA and CA. Based on the clinical outcomes, IA was a better approach than CA. Patients who have undergone IA were less likely to have anastomotic leakage and anastomotic stenosis and had a lower reoperation rate and 90-day mortality rate than CA from the forest plots. While no significant difference was found in pneumonia, in-hospital mortality and 30-day mortality. Anastomotic approaches were usually determined by tumor locations. Accordingly, upper-third esophageal cancer will more likely be handled by the CA approach due to its special location, but the operation approach for the middle-third or lower-third esophageal cancer is usually decided by surgeons. As the primary outcome reported, patients with IA had a lower anastomotic leakage rate. The result was in line with the current high-quality RCT which paid attention to the difference between the two approaches in minimal invasive esophagectomy [8]. The previous systematic review also mentioned that CA had a higher anastomotic leakage rate than IA [4]. Higher tension and worse vascular supply were the key risk factors of anastomotic leakage in the previous studies [9–11]. Compared to CA, the conduit of IA was shorter, which implied a lower tension and better vascular supply and thus facilitating the healing of anastomosis.

Anastomotic stenosis is also a common post-operation complication of esophagectomy. The incidence of anastomotic stenosis was also lower in the IA group, which may associate with the less blood supply of anastomosis concerning the previous results [9–11].

A detailed comparison was performed to explain the similar short-term mortality rates between the two approaches. The anastomotic leakage was divided into 3 types by ECCG classification [12]. IA has a lower type I and type II anastomotic leakage rate than CA while no significant difference was found in type III between the two approaches. Type III anastomotic leakage was defined as a localized defect requiring surgical therapy by ECCG classification, which meant that patients suffering from type III anastomotic leakage are more likely to be life-threatening, compared to type I and type II anastomotic leakage. Therefore, no significant difference was found in 30-day and in-hospital mortality though a higher anastomotic leak rate was found in CA.

A study in 2015 proposed that 90-day mortality following esophagectomy might have a close connection to the readmission rate and a high risk of early mortality was found in patients admitted within 30 days [13]. As the result showed, CA had higher 90-day mortality than IA, which may be due to its high incidence of anastomotic leakage and anastomotic stenosis and high reoperation rate. Thus, compared to CA, IA is a better anastomotic approach.

In line with the previous studies, our study shows a similar result in the incidence of anastomotic leakage, anastomotic stenosis, pneumonia and 30-day mortality. However, different to the previous study, our study is to investigate the more detailed differences between IA and CA through subgroup analysis. Moreover, our study enhances the level of evidence by involving a novel RCT (van Workum et al. [8]) published in 2021. Finally, our study has a large sample size which includes relevant studies over 20 years, making it more comprehensive and reliable.

However, this meta-analysis also contains some limitations. Firstly, the studies included do not share the same outcomes. Then, the insufficiency of data restricts a further subgroup analysis of the tumor location selection. As the site of anastomosis has a connection to the location of the tumor, the result might be more accurate if the tumor location could be fixed. Furthermore, the variance between the collection criteria concerning each study and its connection towards the anastomotic leakage were lacking in our study. Finally, most of the included studies are cohort studies, which leads to an expected heterogeneity of data. More high-quality studies are needed to verify and update our findings. Future study will focus on the long-term outcomes between IA and CA and the connection between anastomotic technique and anastomotic leakage.

## Conclusion

In conclusion, based on the meta-analysis, IA might be a better anastomotic approach than CA. A lower incidence of anastomotic leakage and anastomotic stenosis was found in IA group and no increase in short-term mortality was indicated. Although heterogeneity and publication bias might limit the reliability of the results, surgeons should make a more cautious judgement of the operation approaches.

## Data Availability

All data generated or analyzed during this study are included in this published article.

## Abbreviations

CA: Cervical anastomosis
IA: Intrathoracic anastomosis
RCT: Randomized controlled trial
OR: Odds ratio
CI: Confidence intervals

## References

[1] Sung H, Ferlay J, Siegel RL (2021). Global Cancer Statistics 2020: GLOBOCAN Estimates of Incidence and Mortality Worldwide for 36 Cancers in 185 Countries. CA Cancer J Clin.

[2] Chang AC (2013). Incisions and esophagectomy: is surgical approach all that matters?. JAMA Surg.

[3] Barbat J (1913). Thoracic Esophagectomy: Report of a Case. California state journal of medicine.

[4] Deng J, Su Q, Ren Z (2018). Comparison of short-term outcomes between minimally invasive McKeown and Ivor Lewis esophagectomy for esophageal or junctional cancer: a systematic review and meta-analysis. Onco Targets Ther.

[5] Liberati A, Altman D, Tetzlaff J (2009). The PRISMA statement for reporting systematic reviews and meta-analyses of studies that evaluate healthcare interventions: explanation and elaboration. BMJ (Clinical research ed).

[6] Jadad A, Moore R, Carroll D (1996). Assessing the quality of reports of randomized clinical trials: is blinding necessary?. Control Clin Trials.

[7] Stang A (2010). Critical evaluation of the Newcastle-Ottawa scale for the assessment of the quality of nonrandomized studies in meta-analyses. Eur J Epidemiol.

[8] van Workum F, Verstegen MHP, Klarenbeek BR (2021). Intrathoracic vs cervical anastomosis after totally or hybrid minimally invasive esophagectomy for esophageal cancer: a randomized clinical trial. JAMA Surg.

[9] Okata Y, Maeda K, Bitoh Y (2016). Evaluation of the intraoperative risk factors for esophageal anastomotic complications after primary repair of esophageal atresia with tracheoesophageal fistula. Pediatr Surg Int.

[10] Urschel J (1995). Esophagogastrostomy anastomotic leaks complicating esophagectomy: a review. Am J Surg.

[11] Ohi M, Toiyama Y, Mohri Y (2017). Prevalence of anastomotic leak and the impact of indocyanine green fluorescein imaging for evaluating blood flow in the gastric conduit following esophageal cancer surgery. Esophagus.

[12] Low DE, Alderson D, Cecconello I (2015). International Consensus on Standardization of Data Collection for Complications Associated With Esophagectomy: Esophagectomy Complications Consensus Group (ECCG). Ann Surg.

[13] Hu Y, McMurry TL, Stukenborg GJ, Kozower BD (2015). Readmission predicts 90-day mortality after esophagectomy: Analysis of Surveillance, Epidemiology, and End Results Registry linked to Medicare outcomes. J Thorac Cardiovasc Surg.

[14] Schilling M, Eichenberger M, Wagener V, Stoupis C, Büchler M (2001). Impact of fundus rotation gastroplasty on anastomotic complications after cervical and thoracic oesophagogastrostomies: a prospective non-randomised study. Eur J Surg.

[15] Blewett C, Miller J, Young J, Bennett W, Urschel J (2001). Anastomotic leaks after esophagectomy for esophageal cancer: a comparison of thoracic and cervical anastomoses. Ann Thor Cardiovasc Surg.

[16] Walther B, Johansson J, Johnsson F, Von Holstein CS, Zilling T (2003). Cervical or thoracic anastomosis after esophageal resection and gastric tube reconstruction: a prospective randomized trial comparing sutured neck anastomosis with stapled intrathoracic anastomosis. Ann Surg.

[17] Homesh N, Alsabahi A, Al-Agmar M (2006). Transhiatal versus transthoracic resection for oesophageal carcinoma in Yemen. Singapore Med J.

[18] Okuyama M, Motoyama S, Suzuki H, Saito R, Maruyama K, Ogawa J (2007). Hand-sewn cervical anastomosis versus stapled intrathoracic anastomosis after esophagectomy for middle or lower thoracic esophageal cancer: a prospective randomized controlled study. Surg Today.

[19] Egberts JH, Schniewind B, Bestmann B (2008). Impact of the site of anastomosis after oncologic esophagectomy on quality of life–a prospective, longitudinal outcome study. Ann Surg Oncol.

[20] Kawoosa NU, Dar AM, Sharma ML (2011). Transthoracic versus transhiatal esophagectomy for esophageal carcinoma: experience from a single tertiary care institution. World J Surg.

[21] Klink C, Binnebösel M, Otto J (2012). Intrathoracic versus cervical anastomosis after resection of esophageal cancer: a matched pair analysis of 72 patients in a single center study. World J Surg Oncol.

[22] Zhai C, Liu Y, Li W (2015). A comparison of short-term outcomes between Ivor-Lewis and McKeown minimally invasive esophagectomy. J Thorac Dis.

[23] Huang HT, Wang F, Shen L, Xia CQ, Lu CX, Zhong CJ (2015). Clinical outcome of middle thoracic esophageal cancer with intrathoracic or cervical anastomosis. Thorac Cardiovasc Surg.

[24] van Workum F, van der Maas J, van den Wildenberg FJ (2017). Improved functional results after minimally invasive esophagectomy: intrathoracic versus cervical anastomosis. Ann Thorac Surg.

[25] Liu YJ, Fan J, He HH, Zhu SS, Chen QL, Cao RH (2018). Anastomotic leakage after intrathoracic versus cervical oesophagogastric anastomosis for oesophageal carcinoma in Chinese population: a retrospective cohort study. BMJ Open.

[26] Gooszen JAH, Goense L, Gisbertz SS, Ruurda JP, van Hillegersberg R, van Berge Henegouwen MI (2018). Intrathoracic versus cervical anastomosis and predictors of anastomotic leakage after oesophagectomy for cancer. Br J Surg.

[27] Shao L, Ye T, Ma L (2018). Three-field versus two-field lymph node dissection for thoracic esophageal squamous cell carcinoma: a propensity score-matched comparison. J Thorac Dis.

[28] Schroder W, Raptis DA, Schmidt HM (2019). Anastomotic techniques and associated morbidity in total minimally invasive transthoracic esophagectomy: results from the EsoBenchmark Database. Ann Surg.

[29] Chidi AP, Etchill EW, Ha JS (2020). Effect of thoracic versus cervical anastomosis on anastomotic leak among patients who undergo esophagectomy after neoadjuvant chemoradiation. J Thorac Cardiovasc Surg.

[30] van Workum F, Slaman AE, van Berge Henegouwen MI (2020). Propensity score-matched analysis comparing minimally invasive ivor lewis versus minimally invasive Mckeown Esophagectomy. Ann Surg.

[31] Jezerskyte E, Saadeh LM, Hagens ERC (2020). Long-term health-related quality of life after McKeown and Ivor Lewis esophagectomy for esophageal carcinoma. Dis Esophagus.

[32] Takahashi C, Shridhar R, Huston J, Blinn P, Maramara T, Meredith K (2021). Comparative outcomes of transthoracic versus transhiatal esophagectomy. Surgery.

